# Effect of Trolox addition to cryopreservation media on human sperm motility

**Published:** 2012-03

**Authors:** Mohammad Baqer Minaei, Mohammad Barbarestani, Saeid Nekoonam, Mir Abbas Abdolvahabi, Nasrin Takzare, Mohammad Hossein Asadi, Azim Hedayatpour, Fardin Amidi

**Affiliations:** *Department of Anatomy, Faculty of Medicine, Tehran University of Medical Sciences, Tehran, Iran.*

**Keywords:** *Human spermatozoa*, *Trolox*, *Cryopreservation*, *CASA*, *Motility characteristics*

## Abstract

**Background:** Sperm parameters and motion kinetics are affected by cryopreservation.

**Objective:** The main purpose of the current study was to determine the effect of different concentrations of Trolox as an antioxidant to freezing-thawing procedure on human sperm kinematic parameter.

**Materials and Methods:** Semen was collected from 20 normal donors and divided into five aliquots prior to cryopreservation. The first aliquot was analyzed by computer-assisted sperm analysis (CASA). Other aliquots were mixed with cryo-protective agent containing 0, 20, 40, and 80 µmol Trolox and treated samples were cryopreserved in liquid nitrogen. After two weeks samples were thawed and sperm motion kinematics was measured by CASA. Percent motility (Mot), curvilinear velocity (VCL), straight-line velocity (VSL), average path velocity (VAP), linearity (LIN), and amplitude of lateral head displacement (ALH) were compared before and after freeze.

**Results:** Addition of 40µmol Trolox resulted in significantly higher (p<0.05) post thaw VCL, VSL and VAP compared to other groups. Therefore the percentage of post thaw motile spermatozoa were significantly higher (p<0.01).

**Conclusion:** The supplementation of Trolox significantly improved the post-thawed human semen quality, especially progressive motility and average path velocity.

## Introduction

Cryostorage of human spermatozoa is becoming more important because of new clinical needs and current clinical practice: assisted reproduction, preservation of fertility following chemotherapy, radiotherapy or various surgical procedures, and confirmation of seronegativity for sexually transmitted diseases in semen banking (1-3). 

Sperm cryopreservation has revolutionized the field of assisted reproduction, but sperm parameters and motion kinetics are affected by cryopreservation. Freezing and thawing induce major damage to motility and viability (4) of human spermatozoa because extreme fluctuations in osmolality and temperature in sperm lead to formation of intracellular ice crystals (3) and osmotic stress (5). The cell structure of spermatozoa, the plasma membrane, a large number of mitochondria, low cytoplasm, and low antioxidant in sperm cytoplasm make them potentially susceptible to damage from free radicals (6). 

Reactive oxygen species (ROS) which is produced on cold-shock and osmotic stress of this procedure affect sperm organelles. Exposure to high ROS concentrations can result in the disruption of mitochondrial and plasma membranes, cause chromosomal and DNA fragmentation and bring about a reduction in sperm motility (7). It is recognized that human sperm generate ROS in physiologic amounts, which play a role in sperm functions during sperm capacitation, acrosome reaction, and oocyte fusion. However, uncontrolled and too much production of ROS, when it overwhelms the limited antioxidant defences in semen, results in seminal oxidative stress (8). 

The adverse effects of oxidation can be reduced by antioxidants that are present as an element of seminal plasma. An equilibrium between ROS production and seminal antioxidants normally exists; however, special effects of endogenous antioxidants in poor semen samples are often diminished while the concentration of ROS is abnormally high (7). Antioxidants can protect against ROS; specifically, vitamin E (α-tocopherol) can break the covalent links that ROS have formed between fatty acid side chains in membrane lipids, and is one of the main membrane protections against ROS and lipid peroxidation (9). 

Trolox is a cell permeable, water soluble, derivative of vitamin E with potent of antioxidant properties to prevent mediate oxidative stress and apoptosis. Addition of antioxidant (10, 11), Trolox, to cryopreservation medium maybe improve post-thaw parameters of frozen human spermatozoa (7). 

Similarly in some studies addition of lipid and water-soluble antioxidants such as α-tocopherol and Trolox to semen extender in boor, horse (12), and cat (1) before freezing has been shown to have a protective effect on sperm motility (13) by suppressing lipid peroxidation (9). Therefore, vitamin E supplementation of sperm cryopreservation medium may protect spermatozoa from cryopreservation-induced increases in ROS and following loss of motility (7). 

Motility is one of the most important criteria in assessing sperm quality in normal and abnormal semen specimen. Computer-Assisted Sperm Analysis (CASA) is an automated method that can provide specific information on the kinetic of sperm cells with standard manual semen analysis of World Health Organization (WHO). “CASA has been developed to decrease the amount of time spend in sperm observation, reduce intra-observer differences, and improve the accuracy of final results. This method is objective, accurate, and enables quantification of physical components of sperm movement” (14). 

CASA can be successfully used for either fresh or frozen-thawed semen analysis, and it has already proven its effectiveness in measuring sperm motility parameters before and after the freezing-thawing process (15, 16). Furthermore, characteristics of sperm motion determined by CASA after thawing and preparation could predict pregnancy outcome after intrauterine insemination (IUI) from frozen human sperm (15). 

The aim of this study was to determine affect of addition of water soluble vitamin E, Trolox, before freeze to cryopreservation media on human sperm characteristic motion in normal semen specimen. 

## Materials and methods

**Semen collection**

Semen samples were produced by masturbation into sterile containers from 20 healthy volunteers with proved fertility. The mean age was between 22-40 years and all of them were non-smoker. Samples were obtained after an abstinence period of 5-7 days. 

The approval of Tehran Medical University Research Ethical Committee was obtained prior to the study, and all subjects were informed with respect to this study. Samples were allowed to liquefy at 37°C for 30-40 min. Semen of one subject that failed to liquefy in that time period was excluded.

**Semen analysis**

After liquefaction, 5 µl of semen was loaded on a counting chamber (MAKLER COUNTING CHAMBER 0.01 sq mm 10µm deep SEFI-MEDICAL INSTRUMENT). Total sperm count (×10^6^/ml), percentage motility, sperm grading, and sperm motion kinetics was assessed with CASA. Sperm morphology (percentage normal) was assessed according to 1999 WHO guidelines. Semen specimens with abnormal morphology and <1ml volume were excluded. 

The most useful parameters which are automatically assessed by using computer-assisted sperm analysis system are: VCL, curvilinear velocity (μm s^-1^), the sum of the incremental distances moved in each frame along the sampled path divided by the time taken for the sperm to cover the track; VSL, straight line velocity (μm s^−1^), the straight line distance between the start and end points of the track divided by the time taken for the sperm to cover the track; VAP, average path velocity (μm s^−1^), a derived path based on an average number of points percentage of motile sperm (14).

**Freezing and thawing**

Five experimental groups were prepared from liquefied semen samples. Fresh and pre-freezing samples which were analyzed by CASA assigned as group 1. Samples in groups 2 to 5 were immediately mixed with cryoprotective agent (CPA) in a 1:1 ratio and different concentrations of Trolox (0 as a control, 20, 40 and 80 µmol ), respectively. Then cryovials were suspended in liquid nitrogen vapor (10 cm above the level of liquid nitrogen at -80^o^C) for 15 minutes. Then cryovials were plunged into liquid nitrogen (-196^o^C) and stored for further investigation. 

For thawing cryovials were brought to room temperature or to 37^o^C Incubator after two weeks. Then samples diluted in suitable media, M199, in a 1:1 ratio and centrifuge in 200 g to remove the cryoprotectant. After addition of 1ml M199, the pellet was resuspended in medium M199 and incubated in 37^o^C for 30min; motile sperm cells swim-up and float on each vial.

**Assessment of post-thaw motion and kinematics**

To assess post-thaw sperm parameters, 10µl from each sperm swim-upped sample filled in a makler counting chamber and assessed with CASA. Sperm motion parameters and post-thaw sperm kinematics were evaluated using the computer-assisted semen analysis system (Video Test, ltd: version Sperm 2.1© 1990-2004, Russia). Images were digitized and analysed by video test software; five fields were analysed for each sample.

**Statistical analysis**

Statistical significance were evaluated using ANOVA with Student-Newman-Keuls (S-N-K), Duncan, Games-Howell, and were considered statistically significant if p<0.05. Recovery Rate = (Motility after freezing/ Motility before freezing) ×100.

## Results

The effects of addition different concentration of Trolox on post thaw sperm motility are shown in table I. The motility, progressive motility in Trolox supplemented frozen-thawed groups was significantly (p<0.05) higher than control group. In samples that were cryopreserved by 0, 20 and 80µmol Trolox, the percentage motility was significantly lower in the post thaw samples than in 40µmol samples (prefreeze motility: 52.2%±8.5%; post thaw motility: 19.6%±4.3%, p<0.01). 

The post thaw percentages of motile sperm cells were significantly higher in samples processed by addition of 40µmol Trolox. Cryopreservation resulted in a significant reduction in sperm motility parameters (Table I). Reduce in Rapid progressive spermatozoa (grade a) had a mean value of 14.4% before freezing and a mean value of 0.4% after thawing (p<0.05). 

The slow progressive group (grade b) decreased from 37.8% to 5.7% after cryopreservation (p<0.05). Reflecting the total number of motile spermatozoa, grade a plus grade b motility was also considered. This value declined from 52.2% to 6.4% (p<0.01). The non-progressive motile group (grade c) also decreased significantly after thawing (p<0.05). 

According to table II there were statically significant differences in kinematics’ parameters due to the use of Trolox. Furthermore, addition of 40µmol Trolox resulted in a significantly higher post thaw curvilinear velocity, straight-line velocity, and average path velocity.

After freezing and thawing, the rate of immotile spermatozoa increased significantly from 31% to 87.2% (p<0.01). VAP, VSL, VCL, STR, and LIN were decreases after thawing in no treatment groups (Table II). Straight line velocity is a main value to evaluation of freezing affect. If human spermatozoa exposed to cold-shock stress their motion will be changed from progressive to curvilinear and smooth non progressive (Table I). Treatment with Trolox could improve motion characteristics and motility. 

All the changes mentioned above were found to be statistically significant. Increase in immotile spermatozoa rates were compared using nested test. There was strong correlation between these parameters (p<0.05; Table II).

**Table I:** Comparison between companion effects of treatment on the mean percentages of motion parameters in human semen after freezing-thawing and fresh semen

		**Grade a**	**Grade b**	**Grade c**	**Grade d**	**Class-ab**	**Class-abc**	**RR**
Group 1	fresh semen	14.4^a^	37.8^a^	16.7^a^	31.1^d^	52.2^a^	68.9^a^	
Group 2	freezing control	0^b^	1.8^d^	3.8^d^	94.4^a^	1.8^d^	5.6^e^	8^d^
Group 3	with 20µmol Trolox	0^b^	4.1^c^	5.9^c^	90.1^a^	4.5^c^	9.9^d^	14^c^
Group 4	with 40µmol Trolox	1.3^b^	9.8^b^	12.4^b^	76.5^c^	11.1^b^	23.5^b^	34^a^
Group 5	with 80µmol Trolox	0.4^b^	4.1^c^	7.5^c^	87.9^b^	4.5^c^	12^c^	17^b^

Grade a: Fast progressive motility

Grade b: Progressive motility

Grade c: Non Progressive

Grade d: Non motile

Class-ab: progressive motility

Class-abc: total motility

RR: Recovery Rate

Letters similar to each other in each row are not significantly different.

**Table II:** Comparison between companion effects of treatment on the mean percentages of kinematics parameters in human semen after freezing-thawing and fresh semen

		**VAP**	**VSL**	**VCL**	**ALH**	**BCF**	**STR**	**LIN**
Group 1	fresh semen	24^a^	18^a^	53.04±14^a^	1.1	6.3^a^	70.61±43.6^a^	38.2±9^a^
Group 2	freezing control	9.6^d^	9.2^c^	17.7±15.8^d^	0.5	4.2^c^	54.2±44^c^	26.6±2^b^
Group 3	with 20µmol Trolox	12^c^	10^c^	25.14±17.8^c^	0.6	3.6^d^	43.5±45.9^d^	21.6±5^d^
Group 4	with 40µmol Trolox	17^b^	13^b^	45.1±18.1^b^	0.9	5.6^b^	59.8±48.5^b^	26.1±4^b^
Group 5	with 80µmol Trolox	11^c^	8.7^c^	25.2±15.6^c^	0.6	4.7^c^	54.47±42^c^	23.7±6^c^

VAP: velocity of average path

VSL: velocity of straight line

VCL: velocity of curvilinear

ALH: amplitude lateral head

BCF: beat frequency

STR: Straightness= VSL/VAP×100

LIN: Linearity= VSL/VCL×100

Letters similar to each other in each row are not significantly different.

## Discussion

One of the harmful effects of cryopreservation procedure is ROS generation (17). Normally a balance is maintained between the amount of ROS produced and that scavenged. Sperm damage appears when this equilibrium is disturbed. A shift in the levels of ROS towards pro-oxidants in semen can induce an oxidative stress on spermatozoa. In order to scavenge ROS and reduce their destructive action under normal physiological conditions, a complex antioxidant system is present in sperm and seminal plasma.

It has been reported that antioxidant supplementation had positive effects on human sperm motility (7,18) and mitochondrial membrane potential after thawing (6). The most frequently reported unfavorable effect of cryopreservation on human spermatozoa is a marked decrease in motility (16, 19, 20). It occurs despite many advances in freezing-thawing methodology. There is a remarkable reduce in viability caused by ROS resulting in low conception rate following the use of frozen-thawed sperm for artificial insemination (20). However, sperm viability and motility depends on the cryoprotectant media, freezing technique, oxygen concentration, and presence of antioxidant enzymes in sperm. 

Formation of intracellular ice and ROS production are the main cause of cellular damage during cryopreservation (11). Therefore, we used a known antioxidant to minimize the effects induced by sperm freezing-thawing procedure of fertile men. In the present study, the addition of α-tocopherol before freeze improved sperm motility after thawing. Using a water-soluble-α-tocopherol analogue (Trolox) in the freezing extender as an antioxidant increased motility, depending on the concentration of this. Whereas the Trolox could interact with the water-soluble portion of the plasma membrane too, it may be more effective than others. The effect of α -tocopherol may vary with the concentration, at high concentrations; it may act as an oxidation stimulator rather than an antioxidant.

α-tocopherol supplementation provided protection to sperm viability (21) and was able to inhibit lipid peroxidation (18). In Trolox supplemented frozen-thawed groups, almost all sperm motility parameter was significantly better than that of unsupplemented group. Furthermore, among the supplemented groups, the motility was higher in 100 and 200 μM α-tocopherol supplemented groups than in others. Results of the present study were consistent with the previous observations, where supplementation of α-tocopherol to the freezing extender at 200μg/ml prevented oxidative damage and thus enhanced sperm motility (2). Among the antioxidants, especially α-tocopherol can split the covalent links that ROS have formed between fatty acid side chains in membrane lipids. This result indicates that α-tocopherol plays a significant role in reducing motility damage caused by extreme ROS production during cryopreservation. 

It is well known that the cryopreservation process, involving cooling, freezing, and thawing, induces serious detrimental changes in sperm function. The plasma and acrosomal membranes of spermatozoa are considered to be the primary site of these modifications due to thermal, mechanical, chemical, and osmotic stress and are critical for sperm survival (22). 

Specific components of seminal plasma, particularly proteins, are adsorbed onto the surface of ejaculated spermatozoa. Several polypeptides have been reported to bind to the spermatozoa of several species and to play an important role in their fertilizing ability (23). These sperm coating components seem to maintain the stability of the membrane up to the process of capacitation and/or sperm transport in the female genital tract. 

Other components intervene, either directly or indirectly, in mechanisms aimed to prevent damage to the sperm membrane, oxidative stress, and to maintain the stability of the cell membrane against environmental violence. Therefore, plasma membrane protection of spermatozoa is critical for preservation of their fertilizing ability, especially after stressful procedures such as sex-sorting, freezing, and storage (1, 3, 20).

Regarding kinematical parameters, the result of present study showed significant differences among treatment groups (Table II). These results showed that Trolox in dose of 40 µmol affected kinematical parameters significantly. These results were consistent with those of Castellini *et al* (24) study which showed no significant among kinematical parameters by using different α-tocopherol levels. In contrast jeong *et al* reported that addition of α-tocopherol in fresh and stored boar semen samples significantly increased the percentages of motile spermatozoa and sperm velocity (VAP, VSL, and VCL), linearity and straightness in each treatment (25). 

## Conclusion

The study results revealed a significant post thaw decrease in sperm motility compared with prefreeze sample but addition of 40 µmol Trolox before freeze to cryopreservation media improve all human sperm motion kinetics. 
